# Artemisinin Suppressed Melanoma Recurrence and Metastasis after Radical Surgery through the KIT/PI3K/AKT Pathway

**DOI:** 10.7150/ijbs.97341

**Published:** 2025-01-01

**Authors:** Zhiwei Zhou, Mohd Farhan, Xingan Xing, Wenshu Zhou, Ruohong Lin, Shan Zeng, Mengfang Li, Wenhua Zheng

**Affiliations:** 1 Cancer Center and Center of Reproduction, Development & Aging, Institute of Translation Medicine, Faculty of Health Sciences, University of Macau, Taipa, Macau, China.; 2 MOE Frontier Science Center for Precision Oncology, University of Macau, Macau, China.; 3 Guangdong-Hong Kong-Macao Joint Laboratory for New Drug Screening, University of Macau, Macau, China.; 4 Southern Medical University Cancer Institute, Southern Medical University, 1023 Shatai South Road, Guangzhou, Guangdong, China.; 5 Department of Neurosurgery, Affiliated Hospital of Southwest Medical University, 25 Taiping Street, Luzhou, Sichuan, China.

**Keywords:** artemisinin, melanoma, post-surgery model, c-KIT, AKT

## Abstract

Cancer radical surgery is the primary treatment for melanoma, but almost all malignant melanoma patients get recurrence and metastasis after surgery and are eventually dead. This clinical dilemma appeals to better drugs for post-surgery therapy. Artemisinin is a safe and effective antimalarial drug used in the clinic for decades. However, no information is available regarding the effect of Artemisinins on melanoma recurrence and metastasis after tumor excision. In the present study, we established a post-surgery tumor model on balb/c nude mice, and we found that subclinical dosages of Artemisinin significantly blocked recurrence, metastasis, and extended survival time of mice after tumor excision. Similar results were obtained in the *in vitro* experiments with B16 and A375 cell lines. Further experiments confirmed that Artemisinin inhibits melanoma *in vitro* and *in vivo* after radical surgery by the c-KIT/PI3K/AKT signaling pathway. Our findings support the therapeutic potential of Artemisinin in malignant melanoma after surgery.

## Introduction

Malignant melanoma is one of the most fatal cancers worldwide, whose morbidity has increased rapidly these years. The standard therapeutic regimen for most solid tumors, including melanoma, is cancer radical surgery, followed by chemotherapy, radiotherapy, and other adjuvant therapies 1. However, even with thorough treatments like radical surgery, patients always get recurrence, metastasis, and life loss. The 5-year survival rate is less than 50% on average 2, 3 and even less than 10% in stage IV melanoma with metastasis 4. Patients with distant metastasis will suffer from a much poorer prognosis with a median survival time of 6-12 months 5. This incurable disease makes it a great challenge for modern medicine, and researchers are striving to seek new effective therapies for melanoma.

Nowadays, *in vivo* animal models are commonly used in cancer studies, among which the mouse tumor model is the most frequent and important. Several mouse models for solid tumors are used, including spontaneous, carcinogen-induced, transplanted, and transgenic tumors 6, 7. These models are suitable for observing primary tumor proliferation and distant tumor metastasis, but the *in situ* tumor models cannot simulate the clinical situation of tumor excision. Therefore, post-surgery models are urgently needed, which can mimic the clinic better.

Moreover, melanoma is notorious for its phenotypic diversity and heterogeneity. The treatment and prognosis differ significantly from primary melanoma to recurrent and metastatic melanoma patients 4, 8. The molecular characteristics like T cell fraction are also distinct in metastatic recurrence after surgical excision compared to primary melanoma 9, 10. Given that tumor excision plays a pivotal role in melanoma treatment, only based on the post-surgery model may we clarify the mechanism in recurrent and metastatic melanoma and discover better therapy.

Artemisinin, extracted from the Chinese medicine Artemisia annua also named Qinghao, has been applied to treat malarial for decades. Artemisinin is a safe drug with no or slight side effects 11-13. It was first put forward by Chinese scientist Youyou Tu who won the Nobel Prize for Physiology or Medicine in 2015. Recently, Artemisinin and its derivatives (termed artemisinins) have been reported to have a wide range of bioactivities on different diseases or dysfunction other than malaria, such as neurodegenerative disease, virus infection, oxidative damage and cancer 14-16. Some primary studies have reported that Artemisinin and its derivatives are able to inhibit the proliferation and metastasis of melanoma *in vitro* and *in vivo* 17-20. However, no report has studied the anticancer effect of Artemisinin after tumor excision *in vivo*, which should be more persuasive for its clinical application. As reported by the previous studies, Artemisinins exert their anticancer effect through a complicated regulatory network that consists of multiple targets and signaling pathways, including the PI3K/Akt signaling pathway 21-24.

However, how Artemisinins work on the upstream protein/s and lead to the inhibition of the PI3K/Akt pathway is unknown. KIT, also named as c-KIT or CD117, is a member of class III transmembrane receptor tyrosine kinases (RTKs) and the activator of the PI3K/AKT pathway 25, 26. KIT alterations, including mutations and amplification, are critical in malignant melanoma. KIT negatively relates to favorable prognosis in patients, and its inhibitors like Imatinib receive a moderate therapeutic effect on melanoma patients 27-29.

It is unknown whether Artemisinin could inhibit melanoma recurrence or metastasis after tumor excision surgery. Meanwhile, none of the reported studies has noticed the relationship between Artemisinin and KIT. The present study showed that Artemisinin in subclinical dosages significantly blocked the host animal's melanoma recurrence, metastasis, and extended life appetency after tumor excision. We also identified KIT as a new key mediator of Artemisinin. Artemisinin blocked melanoma growth, migration, and invasion by inhibiting the c-KIT/PI3K/AKT signaling pathway.

## Results

### Artemisinin suppressed melanoma *in vivo* in combination with radical cancer excision

**Figure 1:**
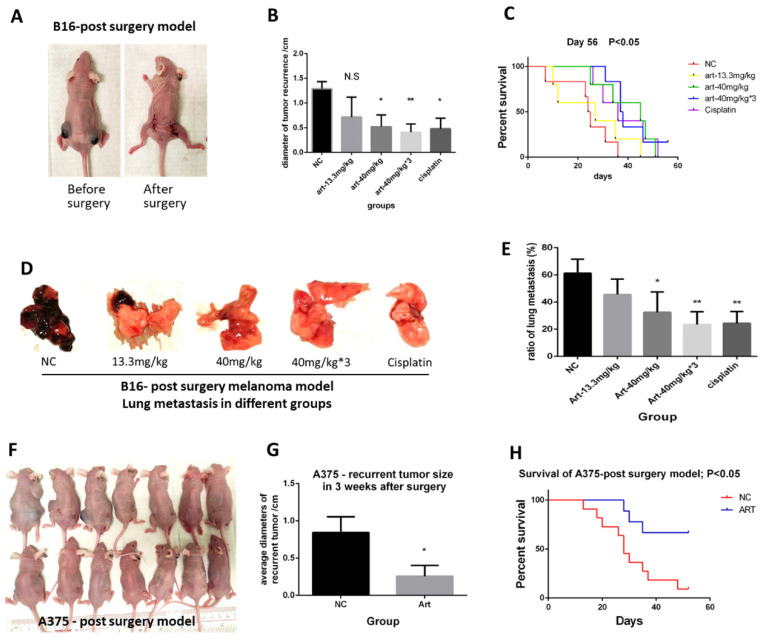
Artemisinin inhibited melanoma growth, recurrence and metastasis, and prolonged survival time. A. Mice before and after the post-surgery model. B. The size of recurrent melanoma three weeks after surgery in 16-induced tumor model. C. Survival analysis in B16-induced post-surgery model between different treatment groups. D. The typical pictures of lung metastasis in B16-induced post-surgery model. E. The ratio of lung metastasis in B16-induced post-surgery model. F. Mice in 3 weeks after surgery in A375-induced post-surgery model between artemisinin (Art) and negative control (NC) groups. G. The statistical result of recurrent tumor size in the two groups. H. Survival analysis in A375-induced post-surgery model. *P<0.05, **P<0.01.

We first illustrated the *in vivo* effect of Artemisinin on inhibiting melanoma. We set up the post-surgery model by performing excision to remove the primary subcutaneous melanoma induced by B16 melanoma cell line (Fig. 1A). After the cancer radical surgery, mice were divided into 5 groups, including the negative control (NC), Artemisinin of 13.3mg/kg per day (Art-13.3mg/kg group), Artemisinin of 40mg/kg per day (Art-40mg/kg group), Artemisinin of 40mg/kg for 3 times per day (Art-40mg/kg×3 group) and Cisplatin group as a positive control, and treated as indicated. The melanoma recurrences, metastasis, and survival time of mice after above treatments were then measured. The results showed that Artemisinin of 40mg/kg, 40mg/kg×3 daily and Cisplatin groups significantly decreased the size of recurrent tumors. The effect of Artemisinin of 40mg/kg was similar to the positive control Cisplatin while that of 40mg/kg×3 daily is the best (Fig. 1B). Survival analysis also showed that mice in art-40mg/kg group and art-40mg/kg×3 group enjoyed a longer survival time than that in NC group (Fig. 1C). When mice died from tumor burden or met the endpoint of the experiment, we dissected the mice to check whether lung metastasis occurred (Supplementary Figure 1B). The results showed that Artemisinin inhibited lung metastasis (Fig. 1D-E).

Meanwhile, we used another melanoma cell line A375 to replicate the results. We found similar trends in late experiments in which Artemisinin significantly decreased the recurrent tumor size (Fig. 1F-G), and extended the life expectancy of the modeled mice (Fig. 1H).

Taken together, Artemisinin could inhibit melanoma growth, recurrence, and metastasis and prolonged survival time after cancer radical excision, whose anti-melanoma effect in subclinical dosage was similar to or even better than cisplatin.

### Artemisinin inhibited melanoma growth, migration and invasion *in vitro*

We also confirmed the *in vitro* effect of Artemisinin on inhibiting melanoma with the use of B16 and A375 cell lines.

**Figure 2:**
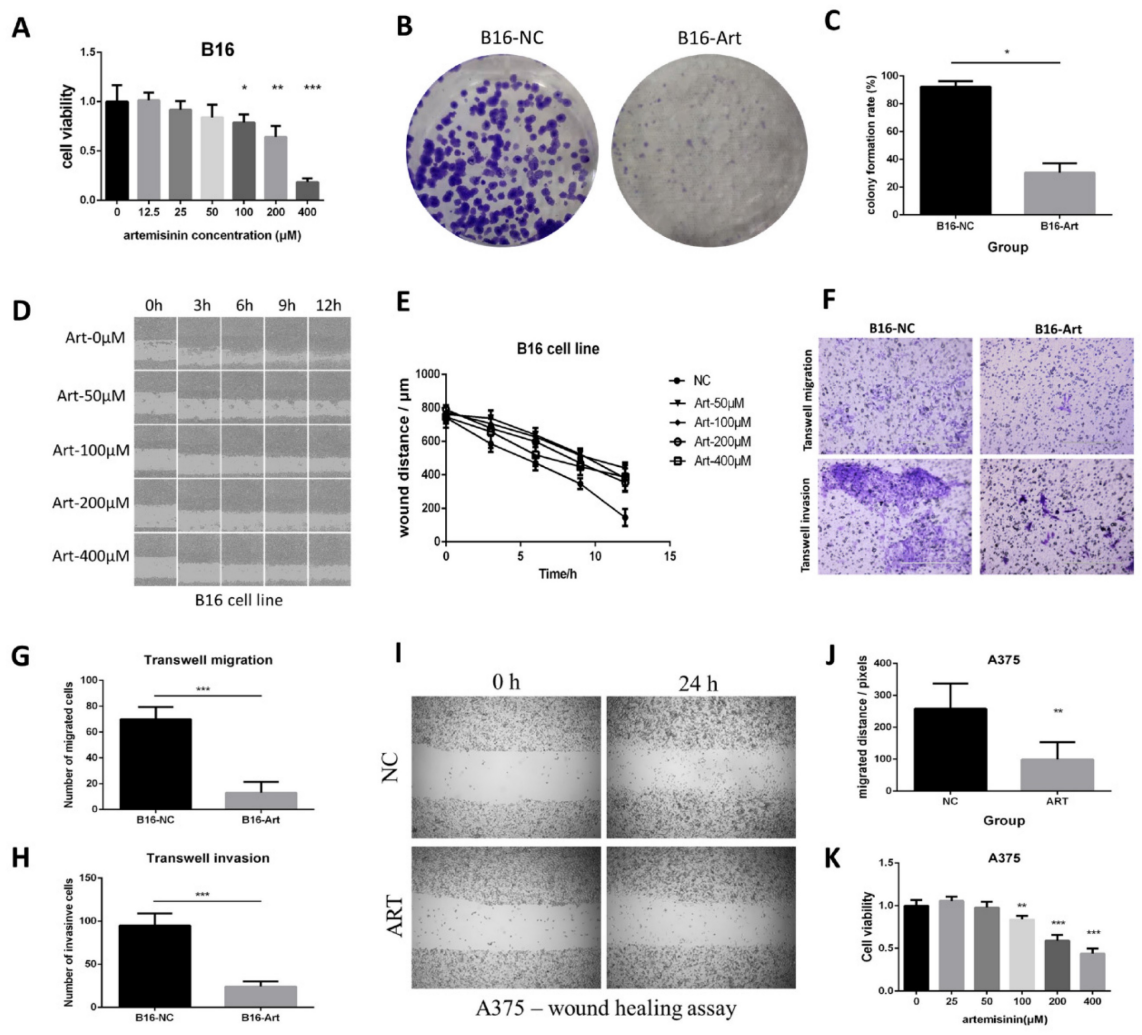
Artemisinin inhibited proliferation, migration and invasion in B16 and A375 cells. A. MTT assay of B16 cells in different groups. B. Colony formation assay in B16 cells. C. Statistical result of colony formation assay in B16 cells. D. Wound healing assay in B16 cell line. E. Statistical result of wound healing assay in B16 cells. F. Transwell migration and invasion assay in B16 cells. G. Statistical result of transwell migration assay in B16 cells. H. Statistical result of transwell invasion assay in B16 cell line. I. Wound healing assay in A375 cells. J. Statistical result of wound healing assay in A375 cell line. K. MTT assay in A375 cells. *P<0.05, **P<0.01, ***P<0.001.

MTT assay was performed on B16 cells to calculate the cell viability in different concentrations of Artemisinin (Fig. 2A), which figured out the IC50 of Artemisinin as 214.89 μM. Therefore, we selected 200 μM of Artemisinin to treat the B16 cells in some of the following experiments. Colony formation assay was carried out to verify that Artemisinin could significantly reduce the proliferation of B16 cells (Fig. 2 B & C). Moreover, we examined the migration ability in different groups by wound healing assay on B16 cell line (Fig. 2D). The result illustrated that Artemisinin (concentration over 50 μM) could significantly decrease the migrated distance of B16 cells compared to the NC group (Fig. 2E). Transwell migration and invasion assays were then performed and the cells that transferred across the Polyester (PET) membrane were imaged and counted (Fig. 2F), which also suggested that Artemisinin could inhibit melanoma migration (Fig. 2G) and invasion (Fig. 2H).

A similar effect was obtained on the A375 cell line. A wound healing assay showed that Artemisinin could significantly inhibit cell migration (Fig. 2I-J). Transwell migration assay and transwell invasion assay further confirmed that Artemisinin significantly decreased the ability of migration and invasion (Supplementary Figure 2 A-B). MTT assay indicated that Artemisinin also suppressed A375 cell proliferation (Fig. 2K) while Colony formation assay also proved the similar trend (Supplementary Figure 2 C-D).

### Pathways screening for Artemisinin derived from RNA sequencing and high throughput analysis

Having assured the anti-melanoma effect of Artemisinin both *in vivo* and *in vitro*, we further explored its mechanism by RNA-sequencing analysis.

**Figure 3:**
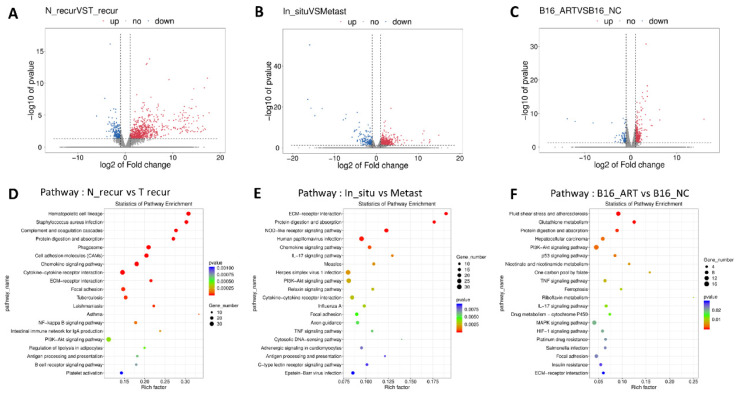
Pathways screening for artemisinin derived from RNA sequencing and high throughput analysis. A. Volcano Plot for differentially expressed genes of recurrent melanoma samples between recurrence-NC and recurrence-Art group. B. Volcano Plot for differentially expressed genes between primary tumor tissues and lung metastasis tissues. C. Volcano Plot for differentially expressed genes of B16 cell line between NC group and Art group. D. Top 20 pathways involving the differential genes between recurrence-NC group and recurrence-Art group. E. Top 20 pathways involving the differential genes between primary tumor and lung metastasis. F. Top 20 pathways involving the differential genes between B16-NC group and B16-Art group.

With the post-surgery model, we acquired some tissues which were named according to their groups as *In_situ* group (primary tumor before surgery), Recurrence-NC group (N_recur group, recurrent tumor after surgery in NC group), Recurrence-Art group (T_recur group, recurrent tumor after surgery in artemisinin-treated group) and lung metastasis group (Metast group). Volcano figures were used to show the differentially expressed genes between the N_recur and T_recur groups (Fig. 3A) and between primary tumor and lung metastasis (Fig. 3B). Meanwhile, the differential genes of *in vitro* treatment were also shown in the volcano figure, including the B16-NC group and B16-Art group (Fig. 3C). Then, the KEEG Pathway analysis based on the above differential genes was carried out, and the top 20 pathways in each analysis were shown (Fig. 3D-F). Several pathways, including the PI3K-AKT pathway, were involved in all three analyses, which suggested that these pathways might be related to melanoma metastasis and artemisinin treatment both *in vivo* and *in vitro*.

### Further screening for potential targets of Artemisinin in inhibiting melanoma

**Figure 4:**
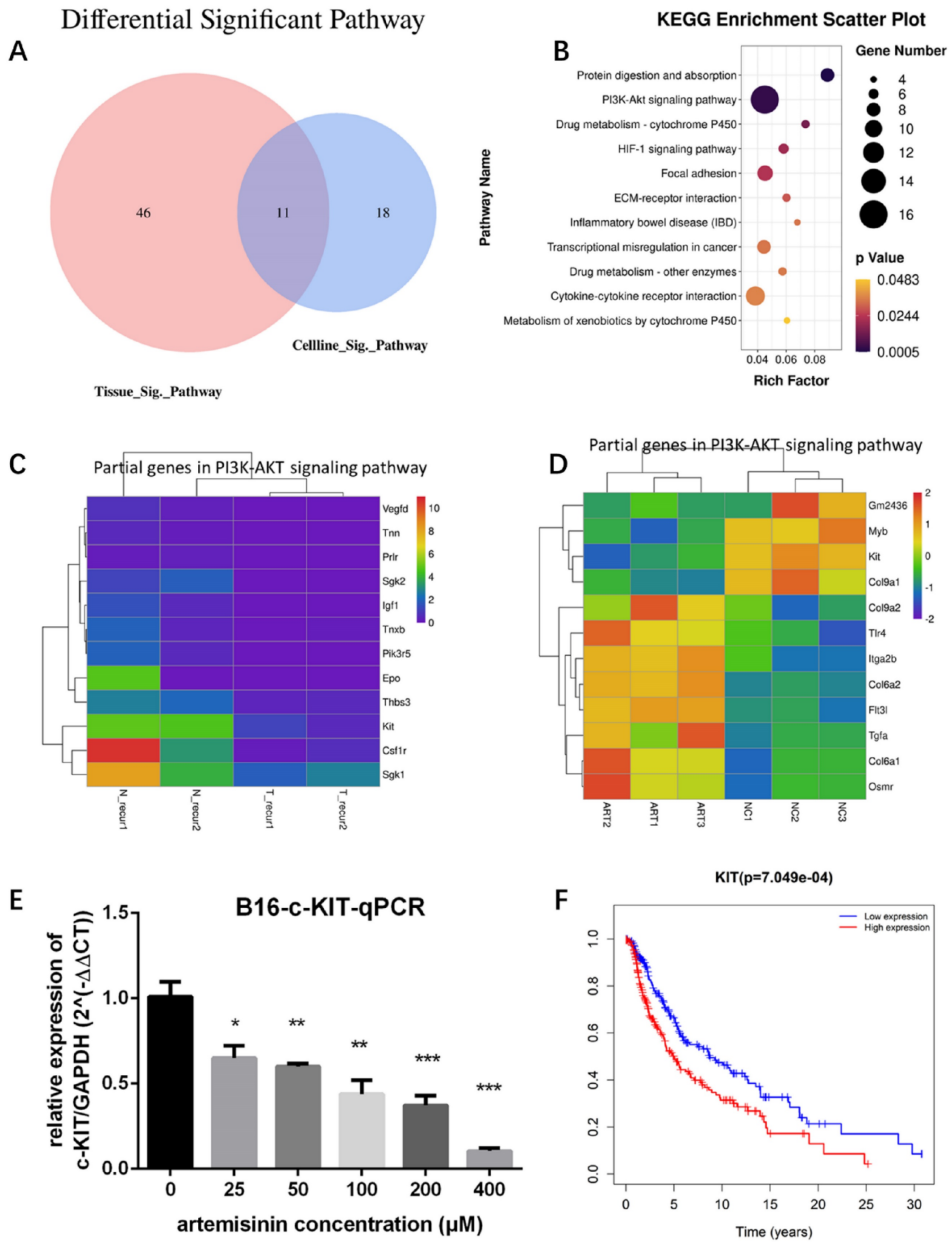
Targets screening for artemisinin with high throughput analysis. A. Venn diagram for Artemisinin-induced significant pathways between melanoma tissues and B16 cells. B. Bubble diagram for the 11 intersectant pathways in artemisinin-treated melanoma tissues and B16 cells, among which PI3K/AKT signaling pathway contained the most gene numbers. C. Heatmap of some differential genes between the recurrence-NC group and recurrence-Art group involved in the PI3K-AKT signaling pathway. D. Heatmap of some differential genes between the B16-NC group and B16-Art group involved in the PI3K-AKT signaling pathway. E. qRT-PCR confirmed c-KIT downregulation by artemisinin in B16 cells. F. The survival analysis of c-KIT based on 458 melanoma patients and related data in the TCGA database.

To identify the significant pathways related to Artemisinin, we drew a Venn diagram to show the intersection of Artemisinin-related pathways between tissue and cell line (Fig. 4A). The intersectant 11 pathways were shown in a Bubble diagram (Fig. 4B), among which the PI3K/AKT signaling pathway contained the most gene numbers. So, we selected this pathway as a potential pathway triggered by Artemisinin. We further figured out the exact differential genes related to Artemisinin involved in the PI3K-AKT pathway (Fig. 4C-D), which indicated that KIT was downregulated both *in vitro* and *in vivo*, suggesting KIT as an important target of Artemisinin. RT-qPCR assay proved that Artemisinin significantly decreased KIT expression in B16 melanoma cell line (Fig. 4E). To confirm the critical role of KIT in melanoma, we carried out the survival analysis based on 468 melanoma patients in TCGA database, which identified KIT as a significant biomarker negatively related to prognosis in melanoma patients (Fig. 4F).

### Artemisinin targeted the c-KIT/PI3K/AKT signaling pathway

**Figure 5:**
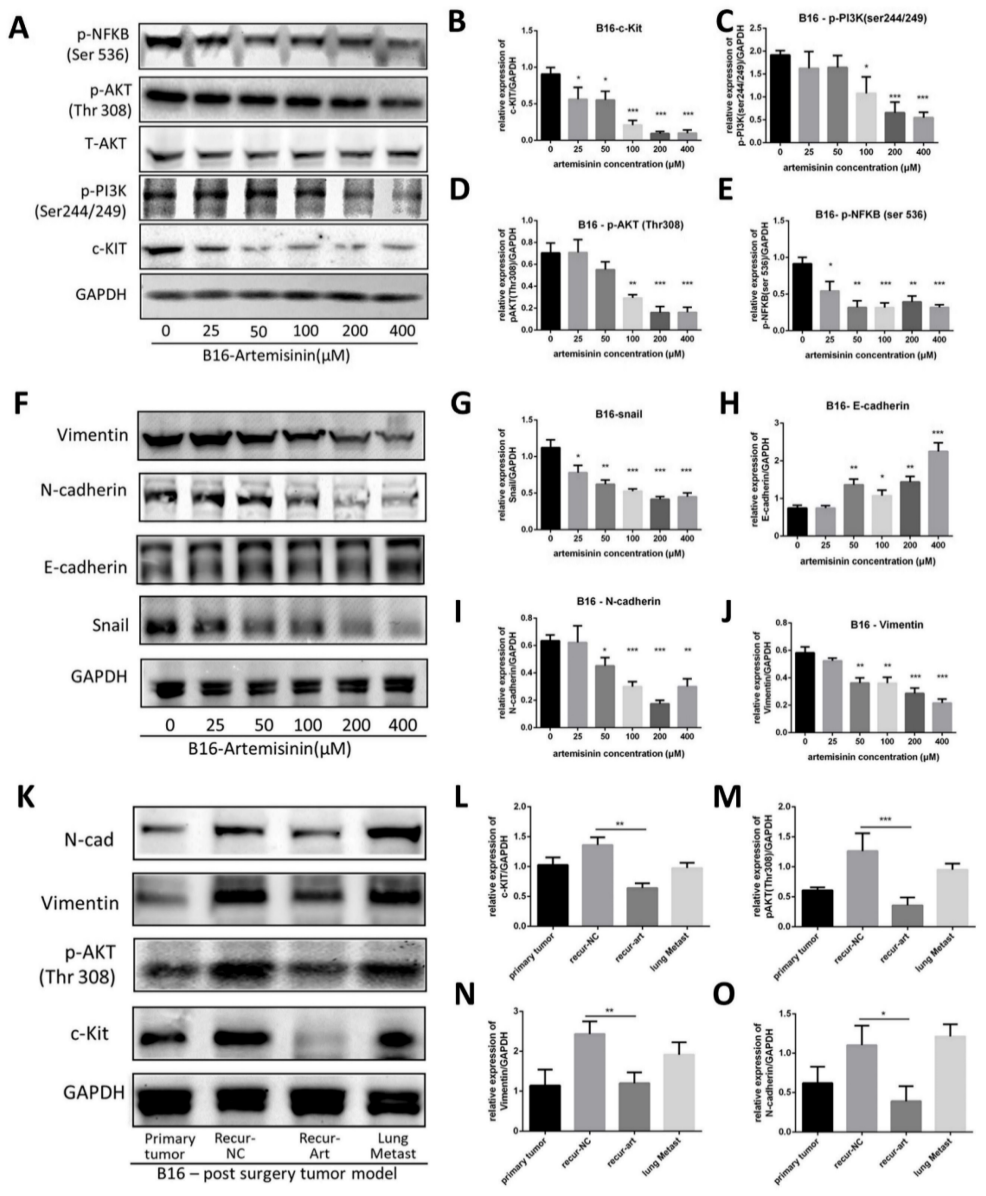
Artemisinin triggered c-KIT induced pathway. A. Western blot for c-KIT, phosphorylated PI3K, AKT and NFKB treated by artemisinin. B. Statistical result of the c-KIT relative expression. C. Statistical result of the relative expression of phosphorylated PI3K. D. Statistical result of the relative expression of phosphorylated AKT. E. Statistical result of the relative expression of phosphorylated NFKB. F. Western blot for Epithelial-Mesenchymal Transition (EMT) markers treated by artemisinin. G. Statistical result of the Snail relative expression. H. Statistical result of the E-cadherin relative expression. I. Statistical result of the N-cadherin relative expression. J. Statistical result of the Vimentin relative expression. K. Western blot for four groups in tissue from the post-surgery model. L-O. Statistical result of the relative expression of c-KIT, phosphorylated AKT, N-cadherin and vimentin in tissue samples. *P<0.05, **P<0.01, ***P<0.001.

We applied Artemisinin to melanoma both *in vitro* and *in vivo*, and then a western blot assay was performed to confirm c-KIT as a target of Artemisinin (Fig. 5A). As a result, Artemisinin significantly decreased c-KIT expression dose-dependently (Fig. 5B). It also attenuated the phosphorylation of PI3K (ser244/249), AKT (Thr 308), and NFKB (ser536). (Fig. 5C-E) suggesting that Artemisinin might regulate the PI3K/AKT signaling induced by c-KIT.

We also investigated the expression of EMT (epithelial-mesenchymal transition) markers regulated by Artemisinin (Fig. 5F). The results illustrated that Artemisinin significantly decreased the expression of Snail, N-cadherin and Vimentin while increasing the expression of E-cadherin (Fig. 5G-J), supporting that Artemisinin was able to suppress EMT to inhibit melanoma migration and invasion.

Similar results were obtained in *in vivo* study where the tissue samples were collected from the post-surgery model for western blot assay, including primary tumor before surgery, recurrent tumor after surgery in NC group, recurrent tumor after surgery in the Art group and lung metastasis. The expressions of c-KIT, phosphorylated AKT, N-cadherin and Vimentin were significantly reduced in the recurrence-Art group compared to the recurrence-NC group (Fig. 5K-O).

These results suggested that Artemisinin could trigger the KIT-induced PI3K-AKT pathway through a phosphorylation cascade on inhibiting melanoma.

### Knockout of c-KIT expression mimicked the effect of Artemisinin

To check the effect of c-KIT, we used the gRNA-Cas9 technique to knockout c-KIT in B16 cells and subsequently explored the effect between c-KIT knockout group (B16-KIT-gRNA) and the wild-type control group (B16-NC).

**Figure 6:**
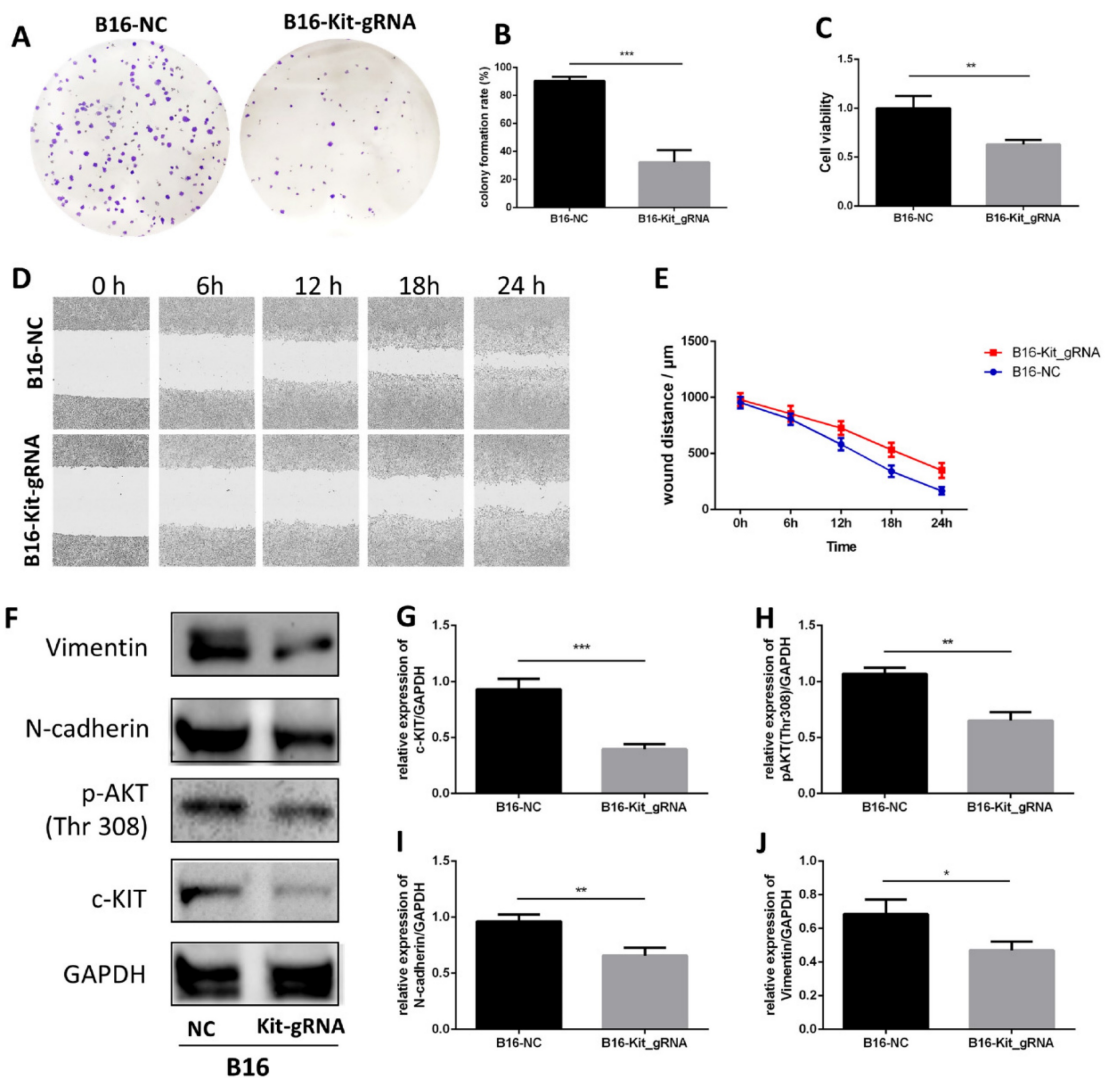
Knockout of c-KIT with gRNA-Cas9 technique significantly inhibits melanoma. A. Colony formation assay between the B16-NC group and B16-KIT-gRNA group. B. Statistical result of the colony formation ratio. C. Statistical result of cell viability derived from MTT assay. D. Wound healing assay between the two groups to detect 24-hour migration. E. Statistical result of the wound distance by time. F. Western blot between B16-NC group and B16-KIT-gRNA group. G. Statistical result of the c-KIT relative expression. H. Statistical result of the relative expression of phosphorylated AKT (Thr308). I. Statistical result of the N-cadherin relative expression. J. Statistical result of the Vimentin relative expression. *P<0.05, **P<0.01, ***P<0.001.

The colony formation assay showed that knockout of c-KIT significantly suppressed cell growth in B16 cell line (Fig. 6A-B). MTT assay also showed a similar trend (Fig. 6C). A wound healing assay was carried out to study the effect of c-KIT knockout in the migration, and the result showed that knockout of c-KIT significantly inhibited migration of B16 cells (Fig. 6D-E).

Western blot was performed between the two groups (Fig. 6F). The results confirmed that c-KIT expression was significantly reduced in the B16-KIT-gRNA group compared to the B16-NC group (Fig. 6G). As expected, the phosphorylation of AKT (Thr308), a downstream target of c-KIT, was significantly downregulated by c-KIT knockout (Fig. 6H). Meanwhile, the EMT biomarkers N-cadherin and Vimentin were decreased in B16-KIT-gRNA group (Fig. 6I-J).

These results indicated that the knockout of c-KIT might suppress melanoma growth and migration by inhibiting c-KIT/AKT signaling pathway.

### Overexpression (OE) of c-KIT attenuated the anti-melanoma effect of Artemisinin

To further elucidate the role of c-KIT, B16 cells were transfected with a plasmid that over-expressed KIT. We carried out the rescued experiments, including three groups: negative control (NC) group, artemisinin treatment (Art) group, and artemisinin treatment with KIT overexpression (Art + KIT_OE) group.

**Figure 7:**
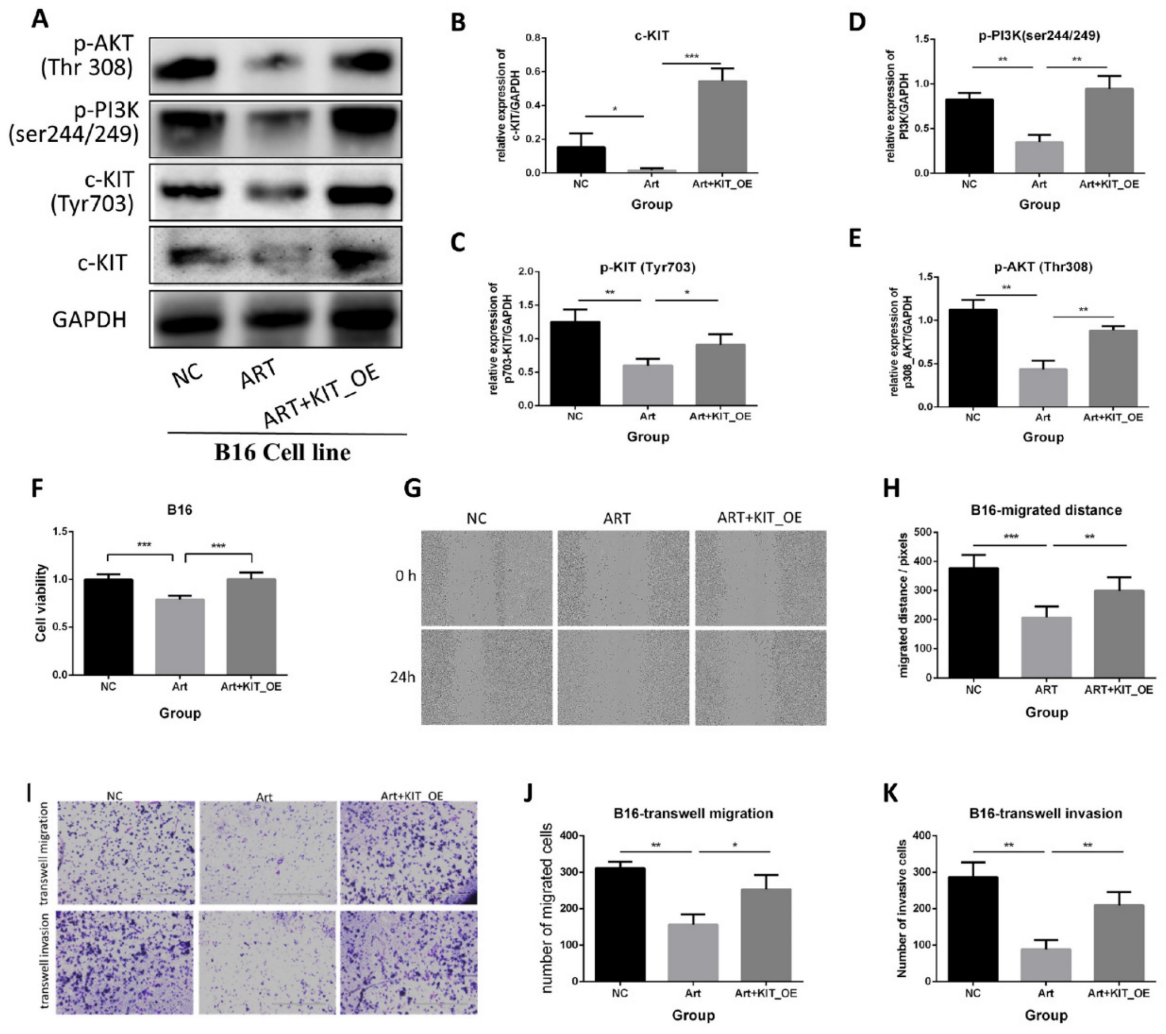
Artemisinin suppressed melanoma by c-KIT related pathway, while rescued experiments with overexpression (OE) of KIT reversed the effects. A. Western blot in NC group, Art group and Art+KIT_OE group. B. Statistical result of the c-KIT relative expression. C. Statistical result of the relative expression of phosphorylated KIT (ser703). D. Statistical result of the relative expression of phosphorylated PI3K. E. Statistical result of the relative expression of phosphorylated AKT (Thr308). F. MTT assay of the three groups in B16 cells. G. Wound healing assay of the three groups in B16 cells. H. Statistical result of wound healing assay. I. Transwell migration and invasion assay for the three groups in B16 cells. J. Statistical result of transwell migration assay. K. Statistical result of transwell invasion assay. *P<0.05, **P<0.01, ***P<0.001.

Firstly, a western blot assay was performed to validate the KIT/PI3K/AKT pathway (Fig. 7A). The results showed that Artemisinin decreased c-KIT expression, phosphorylation, and the phosphorylation of PI3K and AKT, the downstream targets of c-KIT, while overexpression of c-KIT reversed the effect of Artemisinin (Fig. 7B-E). The MTT assay revealed that c-KIT-overexpression reversed the anti-proliferation effect of Artemisinin in B16 cells (Fig. 7F). The wound healing assay disclosed that Artemisinin inhibited B16 migration while c-KIT overexpression reversed the effect of Artemisinin (Fig. 7G-H). The transwell migration and invasion assays had similar results in B16 cells showing that c-KIT-overexpression reversed Artemisinin's effect (Fig. 7I-K).

### The PI3K/AKT pathway inhibitor LY294002 decreased proliferation, migration and invasion in B16 and A375 melanoma cell lines and mimicked the effect of Artemisinin

**Figure 8:**
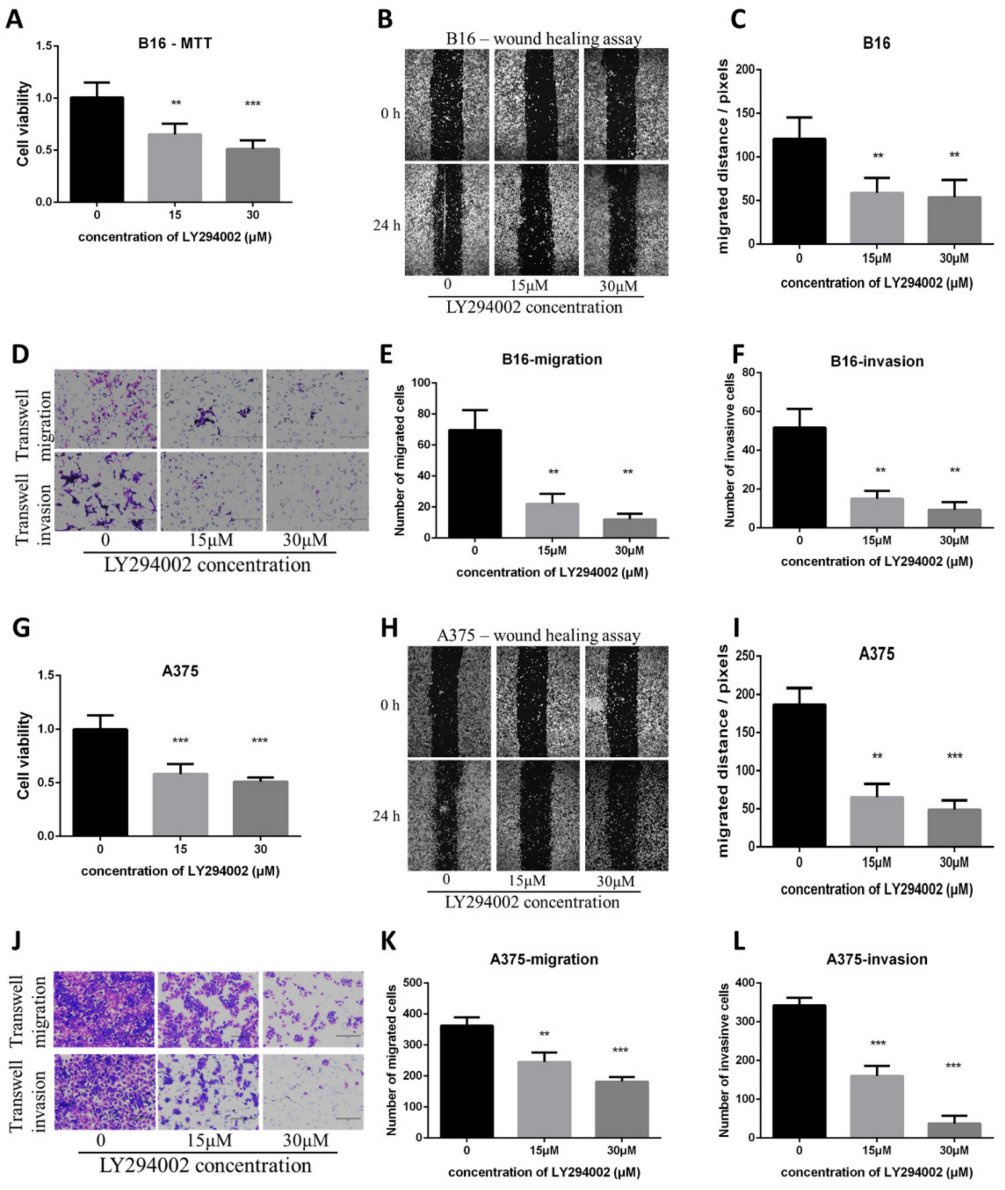
PI3K/AKT inhibitor LY294002 decreased proliferation, migration and invasion in B16 and A375 melanoma cell lines. A. MTT assay among the NC group, LY294002-15μM group, and LY294002-30μM group in B16 cells. B. Wound healing assay of the above three groups in B16 cells. C. Statistical result of the wound healing assay in B16 cells. D. Transwell migration assay and transwell invasion assay among the NC group, LY294002-15μM group, and LY294002-30μM group. E. Statistical result of the transwell migration assay in B16 cells. F. Statistical result of the transwell invasion assay in B16 cells. G. MTT assay of A375 cells among NC group, LY294002-15μM group, and LY294002-30μM group. H. Wound healing assay of A375 cells among the three groups. I. Statistical result of the wound healing assay in A375 cells. J. Transwell migration assay and transwell invasion assay of A375 cells among the three groups in A375. E. Statistical result of the transwell migration assay in A375 cells. F. Statistical result of the transwell invasion assay in A375 cells. *P<0.05, **P<0.01, ***P<0.001.

To check if the inhibition of the PI3K/AKT pathway can mimic the effect of Artemisinin, the PI3K/AKT pathway inhibitor LY294002 was used. The MTT, wound healing, and transwell assays showed that the PI3K/AKT pathway inhibitor LY294002 suppressed proliferation, migration and invasion as Artemisinin did (Fig. 8A-F). Similar results were obtained in A375 cell line, another melanoma cell line (Fig. 8G-L).

These results indicated the PI3K/AKT pathway as pivotal targets in suppressing melanoma cell line A375 and B16 *in vitro*.

### AKT activator attenuated the anti-proliferative, anti-migratory and anti-invasive effect of Artemisinin in B16 and A375 melanoma cell lines

**Figure 9:**
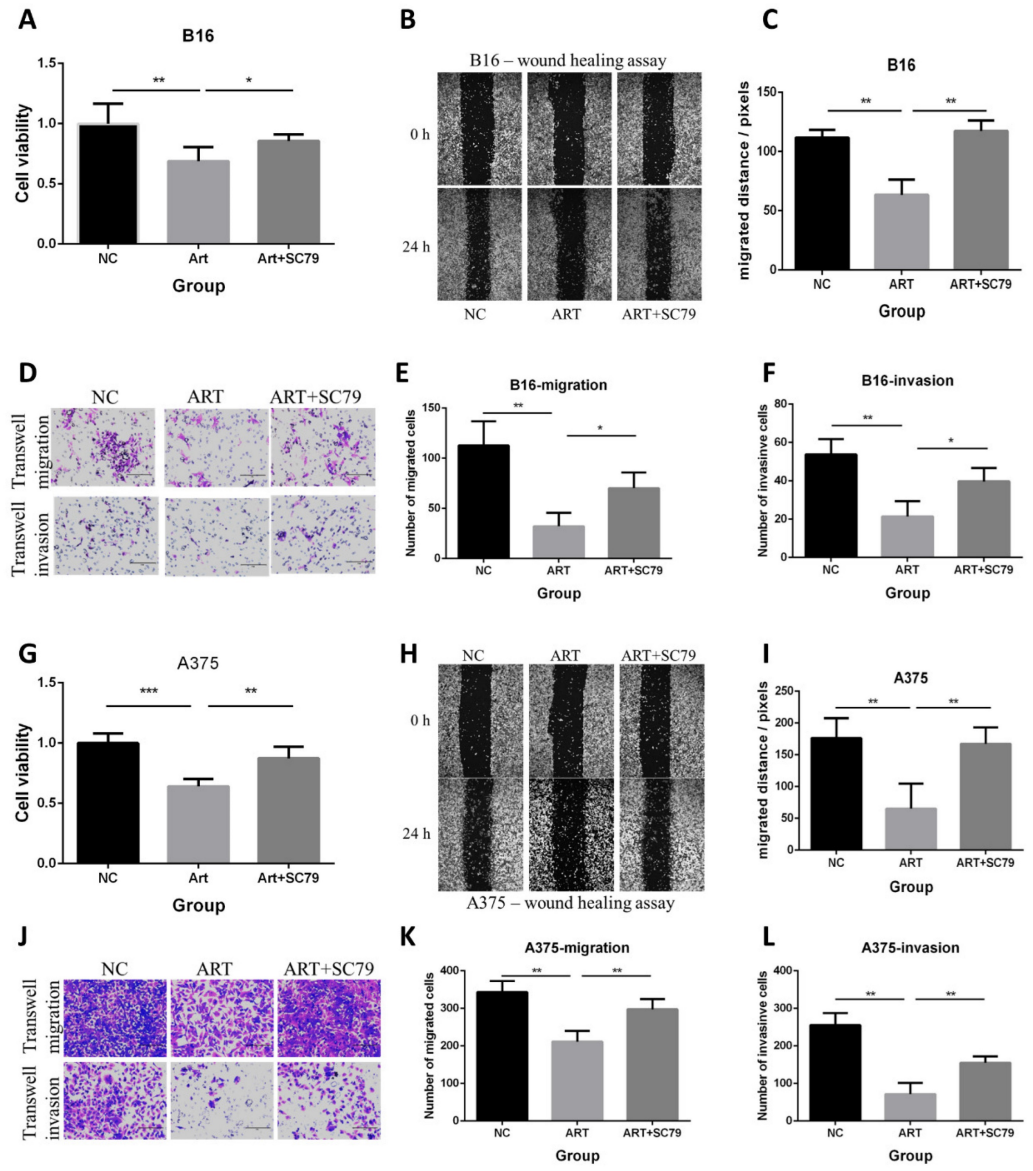
AKT activator SC79 attenuated the anti-proliferative, anti-migratory and anti-invasive effect of Artemisinin in both B16 and A375 melanoma cells. A. MTT assay in B16 cells among the NC group, Art group, and Art+SC79 group. B. Wound healing assay in B16 cells among the three groups. C. Statistical result of the wound healing assay in B16 cells. D. Transwell migration assay and transwell invasion assay in B16 cells among the three groups. E. Statistical result of the transwell migration assay in B16 cells. F. Statistical result of the transwell invasion assay in B16 cells. G. MTT assay in A375 cells among the NC group, Art group, and Art+SC79 group. H. Wound healing assay in A375 cells. I. Statistical result of the wound healing assay in A375 cells. J. Transwell migration assay and transwell invasion assay in A375 cells. E. Statistical result of the transwell migration assay in A375 cells. F. Statistical result of the transwell invasion assay in A375 cells. *P<0.05, **P<0.01, ***P<0.001.

We also used the AKT activator SC79 to further verify the role of AKT in the anticancer effect of Artemisinin in both B16 and A375 cell lines *in vitro*. Three groups of cells included the negative control (NC) group, the Artemisinin (Art) group, and Artemisinin plus SC79 (ART+SC79) group, are set up for the experiment. The MTT assay, wound healing assay, and transwell assay showed that Artemisinin suppressed proliferation, migration and invasion, while AKT activator attenuated the effects both in B16 cells and A375 cells (Fig. 9).

These results further confirm the role of Akt in the anticancer effect of Artemisinin in both B16 and A375 cell lines.

### Overexpression of c-KIT promoted melanoma while the PI3K/AKT inhibitor LY294002 reversed its effect

We further performed a series of rescued experiments to prove the regulatory relationship between c-KIT and PI3K/AKT in B16 melanoma cell line. Western blot showed that overexpression of c-KIT significantly improved phosphorylation of AKT while LY294002 decreased AKT phosphorylation even with c-KIT overexpression (Supplementary Figure 3A-C). Then, a series of *in vitro* experiments indicated that overexpression of c-KIT significantly promoted melanoma proliferation, migration and invasion, while LY294002 reversed the trends (Supplementary Figure 3D-I). These results confirmed that c-KIT could regulate melanoma by AKT phosphorylation.

### Dihydroartemisinin and artesunate suppressed melanoma *in vivo* after radical cancer surgery

We also detected the anti-melanoma effect of two artemisinin derivatives, Dihydroartemisinin (DHA) and artesunate (AS)* in vivo* after cancer radical surgery.

**Figure 10:**
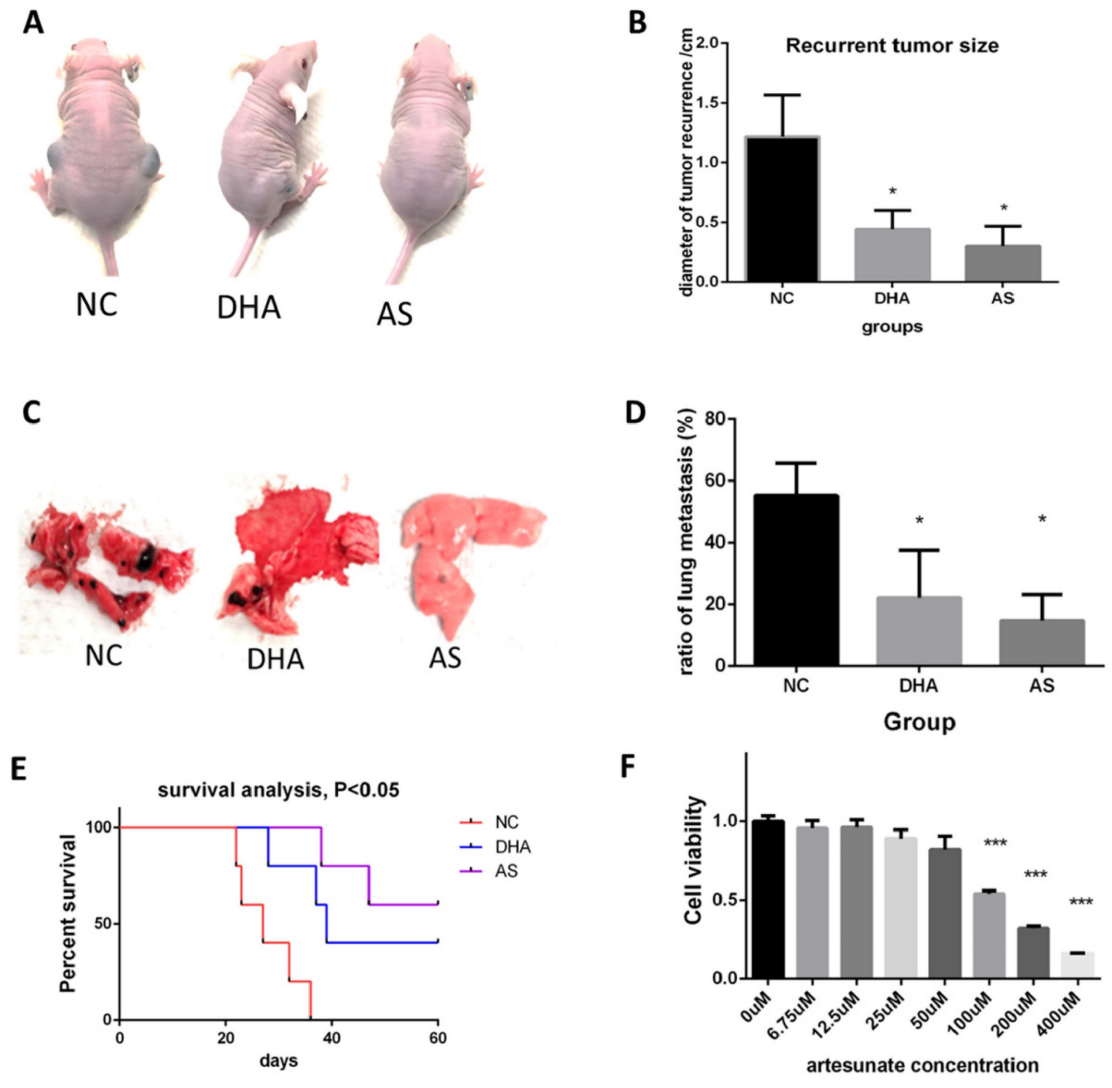
Dihydroartemisinin (DHA) and artesunate (AS) suppressed melanoma *in vitro* and *in vivo* after cancer radical surgery. A. Mice in 3 weeks after surgery in Negatvie control group (NC), dihydroartemisinin treating group (DHA), and artesunate treating group (AS). B. Statistical result of recurrent tumor size in the three groups. C. The typical pictures for lung metastasis in the three groups. D. Statistical result of the ratio of lung metastasis in the three groups. E. Survival analysis among the three groups in 60 days after surgery. F. MTT assay indicated artesunate (AS) inhibited B16 proliferation *in vitro*. *P<0.05, **P<0.01, ***P<0.001.

Like the experiment of Artemisinin, animals were treated as indicated and the melanoma recurrences, metastasis, and survival time of mice were measured. Fig. 10A showed DHA and AS significantly decreased the recurrent tumor within 4 weeks. AS achieved a better therapeutic effect (Fig. 10B). Fig. 10C-D showed that DHA and AS inhibited melanoma metastasis. Survival analysis illustrated that DHA and AS significantly prolonged the survival time for mice in the post-surgery model (Fig. 10E). The *in vitro* effect of AS was also confirmed by MTT, which proved that AS significantly inhibited B16 cell line growth (Fig. 10F).

We further compared therapeutic effects and possible side effects between artemisinin and its derivatives both *in vivo* and *in vitro*. Before, we had proved the effects of ART, DHA and AS respectively in post-surgery model. Now, we compared their effects on inhibiting recurrent melanoma in the same batch of mouse models. Similarly, all the drugs (including ART, DHA and AS) were intraperitoneally injected into mice of post-surgery at a dose of 40mg/kg, with 10% DMSO as negative control (NC). Compared to NC group, DHA and AS presented similar trends that were more significantly than ART on inhibiting melanoma recurrence after surgery, but the difference between ART and DHA/AS showed no significance (Supplementary Figure 4A-B). The change of mice weight after surgery appeared no significant difference among all the groups, suggesting tolerable side effects induced by ART or its derivatives (Supplementary Figure 4C). The *in vitro* MTT assay showed that DHA and AS had similar anti-proliferation effect in B16 cell line compared to cisplatin positive control, which was better than ART (Supplementary Figure 4D). On the other hand, the combination of ART (200μM) and cisplatin (100μM) could further inhibit melanoma growth compared to single therapy of ART (Supplementary Figure 4E), indicating a synergistic effect between ART and cisplatin.

### Artemisinin suppressed breast cancer and lung cancer *in vivo* after cancer radical surgery

To study the anticancer effect of Artemisinin after cancer radical surgery in other cancers, breast cancer and lung cancer were used in the similar experiments.

**Figure 11:**
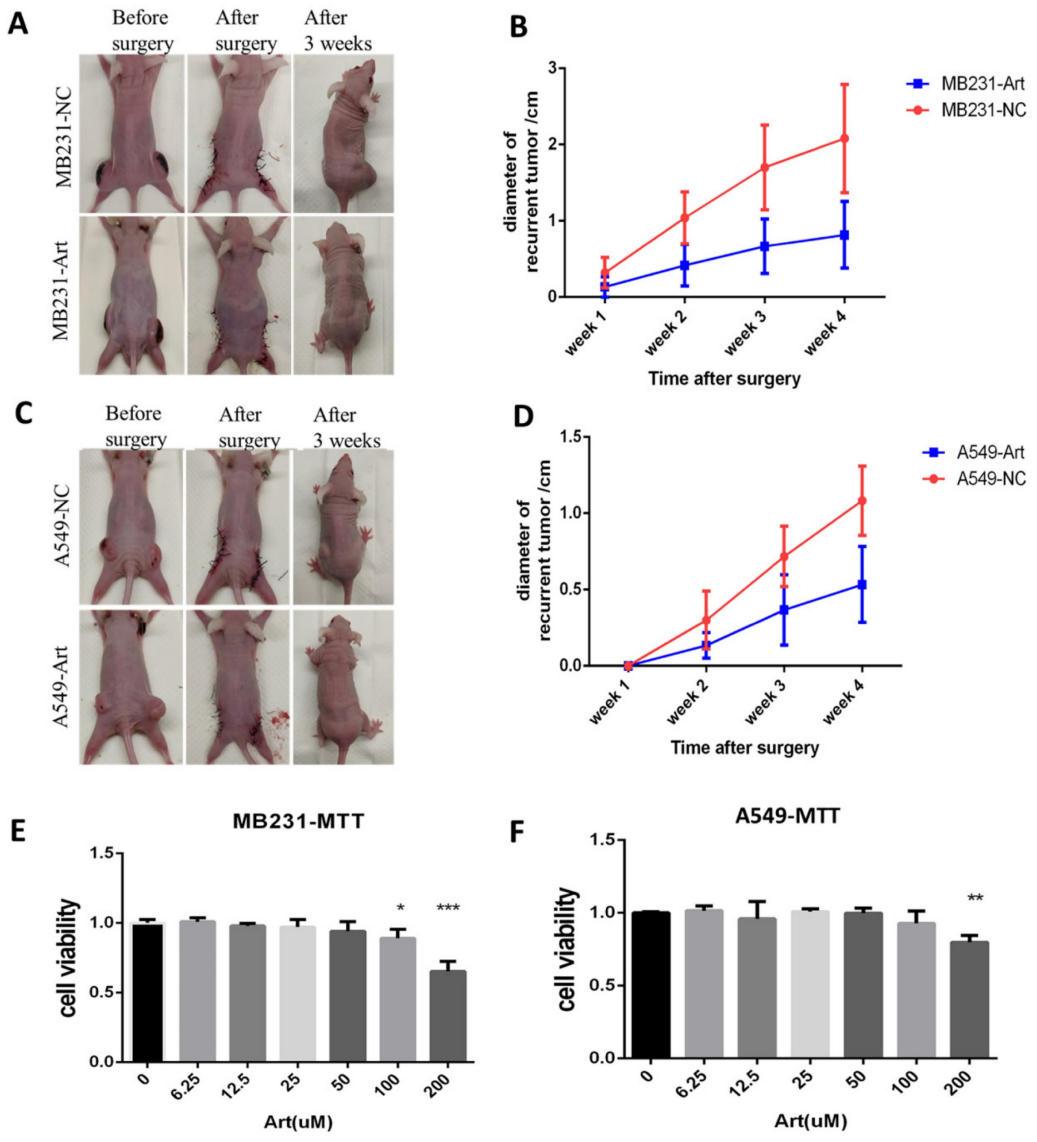
Artemisinin also suppressed breast and lung cancer *in vitro* and in post-surgery mice model. A. Typical pictures of mice in post-surgery model induced by breast cancer cell line MB231 in negative control (NC) group and artemisinin (Art) group. B. Statistical result of recurrent breast cancer after surgery. C. Typical pictures of mice in post-surgery model induced by lung cancer cell line A549 in NC group and Art treatment group. D. Statistical result of recurrent lung cancer after surgery. E. MTT assay of MB231 cells treated by a dose of artemisinin. F. MTT assay of A549 cells treated by a dose of artemisinin. *P<0.05, **P<0.01, ***P<0.001.

We established the post-surgery tumor model for breast cancer with MB231 cell line while for lung cancer with A549 cell line (Fig. 11A). We detected the size of the recurrent tumor in different groups 3 weeks after the surgery for MB231-induced tumor. As a result, Artemisinin significantly decreased the recurrent tumor within 4 weeks compared to the negative control group (Fig. 11B). Similar results were confirmed in the post-surgery model induced by lung cancer cell line A549 (Fig. 11C-D). Besides, the *in vitro* MTT assays also showed that Artemisinin suppressed MB231 cell line and A549 cell line growth *in vitro* (Fig. 11E-F).

## Discussion

The present study creatively established a post-surgery tumor model with mice to simulate clinical cancer therapy after tumor excision. Our research showed that Artemisinin and its derivatives Dihydroartemisinin and Artesunate suppress cancers such as melanoma, breast cancer, and lung cancer, especially melanoma growth, recurrence, and metastasis, and prolong the survival lifespan through the KIT/PI3K/AKT signaling pathway after radical cancer surgery.

Malignant melanoma is one of the fatal cancers, with excision surgery as the primary therapy 1. However, melanoma patients always get a recurrence or/and metastasis and die even if the primary cancer was removed with cancer radical surgery, leading to an overall survival rate of less than 5% in the past decade 30. The treatments following surgery are far from satisfaction, which appeals to new drugs for post-surgery treatment. Nevertheless, few cancer studies have involved an animal model that can simulate radical cancer surgery. The general subcutaneous or other *in situ* tumor models can only disclose the therapy effect on inhibiting primary tumor growth and metastasis, not post-surgery recurrence or metastasis, the major conundrum for clinicians. Under this circumstance, we creatively put forward a tumor-postoperative mice model that not only unveils the potential of Artemisinin in treating melanoma and other cancers after cancer radical surgery, but also enlightens future cancer studies.

No study has been reported about the effect of Artemisinin and its derivatives (ARTs) on tumor recurrence, metastasis, and survival after cancer radical surgery, although some studies about ARTs on primary tumors exist. The effect of ARTs on tumor growth without surgery and tumor growth after cancer radical surgery looks similar but actually much different. For example, our studies in subcutaneous tumor models without surgery and with surgery showed that Artemisinin could suppress melanoma growth to some extent but could not contribute to mice's lifespan in subcutaneous tumor models without surgery. When it came to the post-surgery model, Artemisnin inhibited melanoma recurrence and metastasis better and significantly improved the overall survival time. Moreover, our RNA sequencing data also showed the different gene phenotypes between primary melanoma and recurrence, indicating the different mechanisms between primary melanoma and recurrence after surgery. Consistent with this, a former study has revealed the different risk factors and clinical outcomes between primary and recurrent melanoma 31, suggesting the different mechanisms between tumor growth without surgery and tumor growth after cancer radical surgery. It has also been reported that adjuvant targeted therapy and adjuvant immunotherapy benefit more on high-risk resected melanoma patients 4. These studies highlight the importance and significance of post-surgery model in melanoma mechanism and treatment research.

For malignant melanoma, systemic treatments or drug therapies are also important beyond surgery therapy. The current first-line standard of drug therapies are immunotherapies like PD-1 blockade combined with CTLA-4 blockade, and targeted therapies like BRAF or MEK inhibition 32. These therapies have shown improved survival compared with chemotherapy like cisplatin. However, even with the first-line therapies, most malignant melanoma patients will still succumb to melanoma recurrence and metastasis, indicating the limitations of these therapies. Thus, we need to search for more potential drugs like artemisinin for melanoma treatments. Compared to chemotherapy, artemisinin is proved to interact with some specific targets, making the treatment more precise for patients and less side-effect. Moreover, the combination of artemisinin and other therapies might present joint synergies in treating melanoma.

One of the limitations of artemisinin treatment is the unclear mechanism on treating melanoma after surgery. It was reported that the inhibition of the PI3K/AKT pathway contributes to the antitumor effect of ARTs on primary tumors 24, 33, 34. However, the upstream protein that links ARTs and the PI3K/AKT pathway is not known. Using this new model and different cell lines, we can show that KIT is an upstream protein of the PI3K/AKT pathway. Thus, the effect of ARTs, at least in part, is mediated by KIT and the KIT/PI3K/AKT pathway based on the following. First, the RNA sequencing results from both *in vivo* and* in vitro* disclosed the potential of c-KIT mediated by Artemisinin. Second, Artemisinin could significantly decrease c-KIT expression both at mRNA level and protein level, proved by the qPCR assay and Western blot assay. Finally, the experiments of c-KIT alteration, including c-KIT knockout and overexpression, confirmed its critical role in Artemisinin's effect on melanoma.

**Figure 12:**
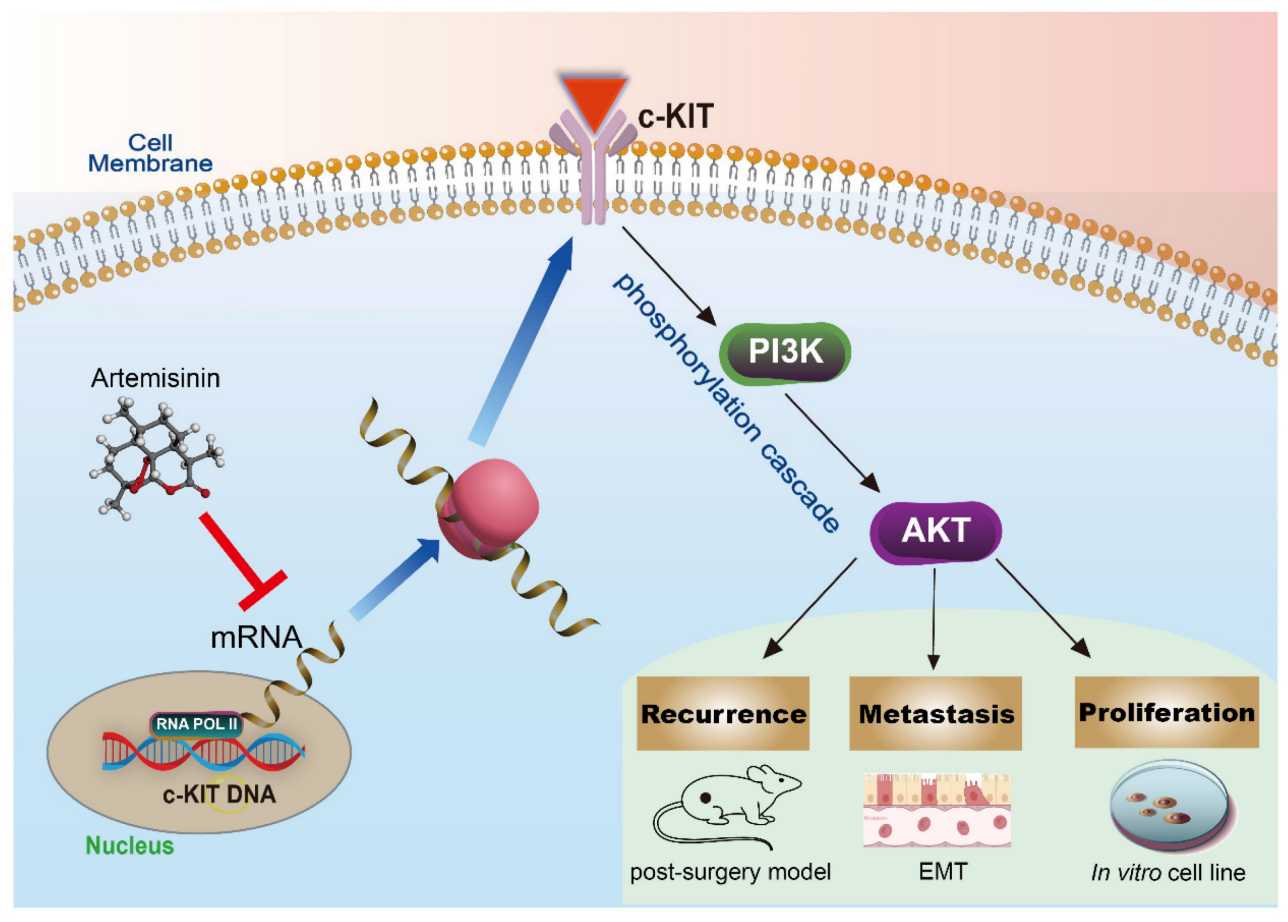
The schematic diagram of the mechanism that Artemisinin can inhibit KIT/PI3K/AKT and EMT signals through phosphorylation cascade to suppress growth, migration, invasion, recurrence and metastasis in melanoma.

KIT is a type III transmembrane receptor tyrosine kinase (RTK) expressing in a variety of cell types, which can regulate the downstream PI3K/AKT pathway through phosphorylation cascade 2, 35, 36. Some articles also pointed out that c-KIT is an oncogenic driver in many cancers, and its mutations and amplifications are crucial to the progress in melanoma 25, 37-39. In the present study, we, for the first time, found c-KIT as a new target of Artemisinin, and further confirmed Artemisinin's effect on regulating the KIT/PI3K/AKT pathway to inhibit the EMT signals and suppressing growth, migration, and invasion of melanoma (Fig.12).

In the past decade, scientists and clinicians have noticed the potential of KIT as a therapeutic target in cancer. Our study has identified it a biomarker that was negatively related to prognosis in melanoma patients. KIT inhibitors, like Imatinib, has been applied clinically in melanoma patients for years, but we still need to explore more drugs targeting c-KIT and other biomarkers, due to the phenotypic plasticity that there are many molecular subtypes and variations in melanoma 2. The KIT inhibitors can only exert their effect in a particular subtype with KIT amplification and specific mutation in melanoma. Thus, we should highlight the combination therapies in future cancer treatments, including radical surgery and targeted therapy like c-KIT inhibitor. Moreover, Artemisinin presented to have more advantage in melanoma therapy, because it could not only regulate c-KIT pathway, but also the complex regulatory networks in cancers as a multi-target drug. ARTs are antimalarial drugs with multiple pharmacological functions on tumor growth and metastasis. Their effect on tumor growth, recurrence, and metastasis after radical cancer surgery in our study may be relative to their multiple targets or effects in addition to the KIT.

As a famous multitarget molecule from Traditional Chinese Medicine, Artemisinin deserves more attention in the treatment of melanoma and other cancers. More importantly, the clinical application of Artemisinin for malarial has proved its safety and tolerability. As reported by John F et. al., artemisinin's derivative AS had been involved in a phase I study in treating advanced solid tumor, which indicated that intravenous AS of 8-25 mg/kg showed modest therapeutic effect with tolerable side effects 40. Based on the equivalent conversion that the drug concentration was consistent per unit body surface area, the drug dose in mice should be about 9 times higher than that in humans, suggesting our equivalent artemisinins dosage might also be potentially safe and effective in clinical therapy. Therefore, our results indicate the potential therapeutic effect of artemisinin in the treatment of cancer patients after radical cancer surgery.

## Conclusion

Artemisinin can suppress melanoma recurrence and metastasis and prolong survival life span after cancer radical surgery in mice models. Artemisinin exerts its anti-melanoma effect through the c-KIT/PI3K/AKT pathway both *in vivo* and *in vitro*, which supports their potential therapeutic effect for post-surgery treatment of cancer patients.

## Methods

### Cell culture

Melanoma cell line B16 and A375 was purchased from the National Infrastructure of cell line resources, China. Cells were cultured in Dulbecco's modified Eagle's medium(DMEM) (Gibico, US) supplemented with 10% Fetal Bovine Serum (FBS) (Gibico, US), 0.1mg/ml streptomycin and 100U/ml penicillin, which were then incubated at 37 ºC humid atmosphere with 5% CO2.

### *In vivo* subcutaneous tumor model

The animal research was approved by Animal Ethics Committee in University of Macau (Number: UMARE-020-2022). Balb/c nude mice (6-8 weeks, half male and female, 23mg on average) were obtained from the Animal Facility, Faculty of Health Science, University of Macau, China. Mice were maintained in Specific Pathogen Free conditions during animal experiments. B16 or A375 cells were cultured and amplified on a 150mm dish, then routinely digested with trypsin, collected into a 15ml tube, and centrifuged at 1000rpm × 5 minutes. Cells were resuspended with DMEM medium and then subcutaneously injected into nude mice's left and right flanks with 5 × 10^6^ cells/200μL per site. The mice's weight and tumor size were monitored and recorded routinely, and the tumor size was estimated by the average diameter L = (a+b) / 2 ("a" was the variable for the major axis, "b" was for the minor axis).

### Post-surgery tumor model based on the subcutaneous tumor model

Based on the subcutaneous tumor model, the post-surgery tumor model was then established. Once the average diameter of subcutaneous tumors grew up to approximately 10 mm, the tumor excision was performed on each mouse. Firstly, mouse was intraperitoneally injected with 1.25% 2,2,2-Tribromoethanol (200μL/10g) for anesthesia before surgery. Then, the mouse was fixed onto the disinfected operation platform to make the subcutaneous tumor well exposed to the operative field. Next, we made a skin incision onto normal skin beside the tumor, separated and removed the tumor as a whole thoroughly. It was important to excise all the affected tissue in this step. Finally, we sutured the incision and put the mouse into postanesthesia recovery.

### Artemisinin treatment for mice *in vivo*

Once the post-surgery melanoma model was established after excluding the mice that died from modeling, the successfully modeled mice were included in the treatment of Artemisinin. All the drugs were dissolved with 10% dimethyl sulfoxide (DMSO) and intraperitoneally injected into each mouse daily for 14 days. The mice were divided into 5 groups, including negative control group treated with solvent DMSO (NC group), Artemisinin of 13.3mg/kg group (ART-13.3mg/kg), Artemisinin of 40mg/kg group (ART-40mg/kg), Artemisinin of 40mg/kg for 3 times per day group (ART - 40mg/kg × 3) and postive control group treated with 10mg/kg cisplatin (Cisplatin group). During and after the treatment, recurrent tumors on some mice were observed and measured. The experiment period was 2 months after the excision surgery. If mice died from melanoma recurrence or metastasis, or met the endpoint of expected period, we sacrificed the mice with carbon dioxide narcosis, dissected the mice and got the samples of recurrent melanoma and the possible lung metastastic tumor, which were stored at -80 ºC fridge or treated with 4% paraformaldehyde (PFA) for further analysis. We would also record the survival time of each mice for survival analysis.

### Paraffin section and HE staining

The animal tissues were previously fixed in 4% paraformaldehyde for 2-5 days, which were then dehydrated with 70%, 85%, 95%, 100% gradient alcohol, and were treated with dimethylbenzene for vitrification. The samples were put into pareffin embedding with Leica EG1150 Embedding Centre (BISC, German) and then slicing with Leica RM2235 Microtome (BISC, German). The dried slices were put into the Leica ST5020-CV5030 Multistainer-Coverslipper (BISC, German) for hematoxylin-eosin (HE) staining, which contained five procedures of dewaxing, dyeing, dehydration, transparency and sealing. Finally, the stained slices were photographed by microscope.

### High-throughput RNA sequencing and further bioinformatic analysis

We carried out high-throughput sequencing assay for tissues and B16 cell lines in different groups, respectively. As for tissue sequencing, there were 4 groups in the analysis, including primary tumor before surgery (In_situ group), recurrent tumor of the negative control group after surgery (N_recur group), recurrent tumor of artemisinin treatment group after surgery (T_recur group) and lung metastasis of negative control group (Metast group). As for cell line sequencing, there were 2 groups, including B16 cells of the negative control group (B16_NC group) and B16 cells treated with 200 μM Artemisinin (B16_ART group). We prepared about 100 mg tissue sample for each case in tissue sequencing and approximately 5×10^7^ cells for each case in cell line sequencing. The samples were sent to Lc-bio Technologies Corporation (Hangzhou, China), where the RNA sequencing would be completed afterward. Then, the gene differential analysis was carried out and the differentially expressed genes were screened out when the absolute value of the logarithm (base 2) of fold change > 1 and p-value < 0.05. The differential genes were put into Gene Ontology (GO), and KEEG Pathway enrichment analysis, and the top 20 pathways with higher gene ratios were selected for further analysis.

### Survival analysis based on TCGA database

We acquired RNA sequencing data and related clinical information of 468 cases of melanoma patients, including primary melanoma and metastatic melanoma, from the Cancer Genome Atlas Program (TCGA database, https://portal.gdc.cancer.gov/). Survival analysis for specific targets among melanoma patients were carried out after the data were processed and standardized with Perl software and R software.

### 3-(4,5-dimethyl-2-thiazolyl)-2,5-diphenyl-2-H-tetrazolium bromide (MTT) assay

Briefly, we seeded cells (1×104) in different groups into 96-well plates that were cultured at 37°C with 5% CO2 atmosphere. After a certain time point, we treated the cells with 0.1% MTT diluted in DMEM medium. After 3 hours, we carefully removed the medium in each well and added 150 μL DMSO to thoroughly dissolve the crystal. Finally, we detected the OD value on 490nm with the use of Microplate Reader to represent the cells proliferation.

### Colony formation assay

Cells (500/well) in different groups were respectively seeded into six-well plates and cultured for about 10 days. Then colonies were fixed for 30 minutes with 4% paraformaldehyde and stained with 0.1% crystal violet for 20 minutes. The number of colonies were photographed and counted while the colony formation rate was calculated to indicate the proliferation.

### Wound-healing assay with Incucyte

Cells were seeded with a density of 1 × 10^5^/well into 96-well plate and cultured to 90% confluence. Each well was pre-treated with DMEM medium without FBS for 12 hours. Then, cell layers were scratched with the sterile wound marker to form wounded gaps. Each well was gently washed with PBS and then cultured in DMEM without FBS. The plate was then put into Incucyte, where the cells were incubated at a suitable atmosphere and each well was auto-photographed every 3 hours. We measured the distance change by time for each wound gap to calculate the migrating ability of cells in different groups.

### Transwell migration and invasion assay

The invasion assay was carried out with the use of transwell inserts (8.0µm, SPL, Japan) coated with 50µL of 1mg/mL Matrigel matrix (BD, USA) diluted by pre-cooling DMEM according to the manufacturer's guideline. After incubated at 37°C for 5 hours, the redundant liquid in each insert was removed, and fresh DMEM medium was added to hydrate the matrigel for 30 minutes. Meanwhile, cells in different groups were routinely collected and resuspended with DMEM without FBS. Next, cells (4 × 10^4^) in 200μl DMEM were seeded into the insert (upper chamber), while 500μL DMEM medium with 10% FBS was added to the 24-well plate where each well (lower chamber) contained an insert. The migration assay was similar to the invasion assay, except that the Transwell insert was not coated with Matrigel.

After incubation for 36 hours at 37°C with 5% CO_2_, cells that did not penetrate the membrane were removed by a cotton swab, while the migrated or invaded cells were fixed with 4% paraformaldehyde, stained with 0.1% crystal violet and photographed with microscope.

### Bacterial culture and competent cell induction

Escherichia coli DH5α was used as the bacterial host that was cultured in lysogeny broth (LB) medium shaking at about 220rpm or on LB agar plate at 37°C incubator. When it was appropriate, antibiotic ampicillin (100mg/ml) was added into the medium for screening the targeted DH5α.

For better transformation to accept plasmid, 50mL DH5α in a suitable concentration was pre-cooled with ice for 10-15 minutes. Then, the tube was centrifuged at 4000g, 4°C for 10 minutes and the sediment was collected, which was subsequently added with 10ml 0.1M CaCl_2_ and treated on ice for 30 minutes. Next, the tube was once more centrifuged with same parameter and the DH5α sediment was collected and treated with 4mL pre-cooled 0.1M CaCl_2_. The competent cells were then well prepared, which could be used for tranformation or stored at -80°C fridge.

### sgRNA design and construction of targeted plasmid

In order to knockout c-KIT gene of B16 cells, sgRNA-CRISPR/Cas9 technique was applied. We designed sgRNA (short guide RNA) of KIT (Mus musculus) according to the instruction of ATUM (https://www.atum.bio/eCommerce/cas9/input). The three pairs of oligos were shown in Table 1, and we selected the third pair for the following experiments based on the knockout efficiency. The oligos were synthesized by BGI (www.bgitechsolutions.cn). Each pair of oligos was annealed in the reaction systemem: 1μL oligo1 (100μM), 1μL oligo2 (100μM), 1μL 10× T4 Ligation Buffer (NEB), 0.5μL T4 PNK (NEB), 6.5μL ddH_2_O. The system was annealed in a thermocycler using the following parameters: 37°C 30minutes, 95°C 5 minutes and then ramp down to 25°C at 5°C/min.

We used a PX459-based plasmid to link the annealed sgRNA into a recombinant plasmid. The construction of backbone plasmid could be seen in Addgene (Plasmid #62988) https://www.addgene.org/62988/. Based on the sequence of backbone plasmid and the designed sgRNA, we were able to used BbsI endonuclease to linearize the plasmid and then link the sgRNA into recombinant plasmid with the use of Quick ligase. The digestion reaction system was: 2 μg PX459 plasmid, 2 μL 10× NEB buffer 2.1, 1 μL BbsI (NEB) and ddH_2_O was added up to 20μL. The reaction was incubated at 37°C for 3 hours. Then, the digested plasmid was purified with the use of Gel Extraction Kit and collected in ddH_2_O. As for the ligation reaction, the reaction system consisted of 2 μL digested plasmid (25ng/μL), 1 μL annealed oligo duplex, 5μL 2× Quickligation Buffer (NEB), 1μL Quick Ligase (NEB) and ddH2O was added up to 11 μL. This system was incubated at room temperature for 10 minutes according to manufacturer recommendations.

Finally, transformation was performed with 4μL lignation product into 50μL competent cells.

### Ligation confirmation and plasmid extraction

After transformation, the HD5α was cultured and amplified in LB medium at 37°C for 1 hour, which was then cultured in LBA with 100mg/mL ampicillin for overnight. Several single clones of bacteria were selected and each one was amplified in LB medium with ampicillin. The sequence of recombinant plasmid was confirmed with the use of Sanger Sequencing by BGI (Beijing, China). The possitive HD5α colony with successful recombinant plasmid was used for plasmid extraction with the use of Pure LinkTM Expi Endotoxin-Free Maxi Plasmid Purification KIT according to the manufacturer instruction.

### Cell line transfection with plasmid

We transfected the purified recombinant plasmid into B16 cells with the use of Lipo3000 (Thermo Fisher, US). We prepared 2 tubes and each tube was filled with 50μL Opti-MEM medium. One was added with 2μL Lipo3000 while the other was added with 4μL P3000 and 2μg plasmid. After 5 minutes, the two tubes were mixed together and further incubated for 15 minutes at room temperature. Cells in plate were washed with PBS and then treated with the mixed medium containing plasmid. After incubation at 37°C for 8 hours, we removed the old medium and added fresh DMEM medium with 10% FBS for another 24 hours culture. Puromycin (2μg/mL) was added to kill the cells that failed to recieve the transfected plasmid. The remaining cells were put to single cell culturing and amplifying. Knockout effect of KIT for each colony was confirmed by qPCR and Western blot.

### Other gene/protein alteration methods

The plasmid (CMV-MCS-EGFP-SV40-Neomycin) with c-KIT overexpression was designed and prepared from Genechem, China (https://www.genechem.com.cn/). The plasimid was transfected into B16 cell line and then neomycin treatment was applied to screen out cells with c-KIT overexpression.

On the other hand, the PI3-kinase specific inhibitor LY294002 (Cat. 440202, Calbiochem, Meck, German) was applied to B16 and A375 cells, which would decrease the PI3K protein and the downstream pathway. The AKT phosphorylation activator SC79 (S7863, Selleckchem, USA) was used in B16 and A375 cells, which would activate AKT phosphorylation.

### Quantitative Real-time Polymerase Chain Reaction (qRT-PCR) analysis

Total RNA was extracted from cells in 6-well plate with 1mL RNAiso Plus (TAKARA, Japan), according to the manufacturer's instruction. The RNA purity and concentration were confirmed by Nanodrop^TM^ One/One^C^ Microvolume UV-Vis Spectrophotometer (Thermo Fisher, US). Reverse transcription was performed to produce complenmentary DNA (cDNA) from total RNA with PrimeScript RT reagent Kit with gDNA Eraser (Cat. RR047A, TAKARA, Japan). The primers were designed as described in Table 2 and synthesized by BGI (Beijing, China). The dye-based quantitative PCR (qPCR) was carried out with iTaq Universal SYBR Green Supermix (Cat. 1725124, Bio-Rad, US) and the procedures were conducted by Bio-Rad^TM^ Real-Time System as below: (1) 95°C for 2min; (2) 45 cycles: denaturation at 95°C for 5 s, annealing and extension at 60°C for 30s with Plate Read at the end-point; (3) melting procedure from 60°C to 95°C with increment of 0.5°C/sec, and with Plate Read for every 5s. The relative expression levels of the PCR products were calculated using the 2-ΔΔCT method.

### Western blot analysis

Protein from cells or tissues were pyrolysed by RIPA Lysis Buffer (Bytotime, China) and denatured at 98°C for 10 minutes. Then the total protein lysates were separated by sodium dodecyl sulfate polyacrylamide gel electrophoresis and transferred to a 0.22 µm polyvinylidene difluoride membrane (Millipore, Billerica, US). Subsequently, the blot was incubated with specific antibodies according to the manufacturer's protocol. The GAPDH antibody was used as the internal preference. The primary antibodies were as follows: GAPDH (SAB, 21612); c-KIT(SAB, 40746); c-KIT (phospho Tyr703) (SAB, 13969); AKT(SAB, 27174); AKT (Phospho-Thr308) (SAB, 11055); PI3 Kinase Class III (Phospho-Ser244/249) (SAB, 13265); NFKB (Phospho-Ser536) (CST, 3033); E-cadherin (SAB, 40860); N-Cadherin (SAB, 48495); Vimentin (CST, 5741); Snail (SAB, 38667).

### Statistical analysis

The significance of differences between groups was estimated by Student's t-test, one-way ANOVAs or Chi-Square test as appropriate. A two-tailed P<0.05 was considered statistically significant. All statistical analyses were performed with SPSS 23.0 software (IBM, SPSS, Chiago, IL, USA).

## Supplementary Material

Supplementary figures.

Supplementary material 1: N_recurVST_recur_Gene_differential_expression.

Supplementary material 2: In_situVSMetast_Gene_differential_expression.

Supplementary material 3: B16_ARTVSB16_NC_Gene_differential_expression.

## Figures and Tables

**Table 1:** The sequence of small guide RNA (sg-RNA) for CRISPR knockout experiment

Name		Sequence
sg-KIT_1	oligo1	CACCGACGTGAAGCGCGCCTACCAC
	oligo2	AAACGTGGTAGGCGCGCTTCACGTC
sg-KIT_2	oligo1	CACCGACACGTAAATAGAACTCGTG
	oligo2	AAACCACGAGTTCTATTTACGTGTC
sg-KIT_3	oligo1	CACCGATCAATGCACGTCAGGCTG
	oligo2	AAACCAGCCTGACGTGCATTGATC

**Table 2:** Sequence of PCR primers

Primer Name	Sequence
Mus-Gapdh-Forward	5'-AAGAGGGATGCTGCCCTTAC-3'
Mus-Gapdh-Reverse	5'-TACGGCCAAATCCGTTCACA-3'
Mus-Kit-Forward	5'-TGTGAACCAACTTCGCCTGA-3'
Mus-Kit_Reverse	5'-GGGCCTGGATTTGCTCTTTGTT-3'
