# Supplementary material for: Artemisinin Suppressed Melanoma Recurrence and Metastasis after Radical Surgery through the KIT/PI3K/AKT Pathway
Source: Int J Biol Sci. 2025 Jan 1;21(1):75–94. doi: 10.7150/ijbs.97341 (PMC11667813; doi:10.7150/ijbs.97341)
Supplement: Supplementary file 1 — Supplementary figures. [file ijbsv21p0075s1.pdf]

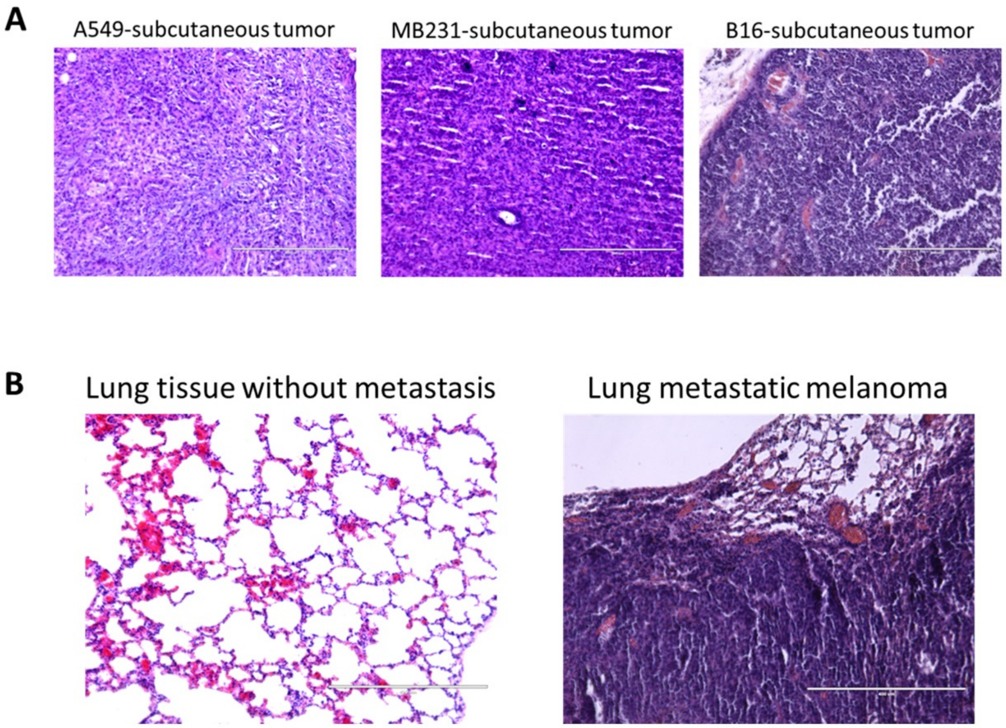

Supplementary Figure 1. HE staining for the primary tumors and lung metastasis. A. After removing primary subcutaneous tumors during post-surgery modeling, the samples were put into HE staining. This picture showed the HE stained subcutaneous lung cancer (derived from A549 cell line), breast cancer (derived from MB231 cell line) and melanoma (derived from B16 cell line). B. This picture showed the HE stained lung tissue without metastasis or with metastatic melanoma. The whole length of the ruler in all the pictures was 400 micrometer.

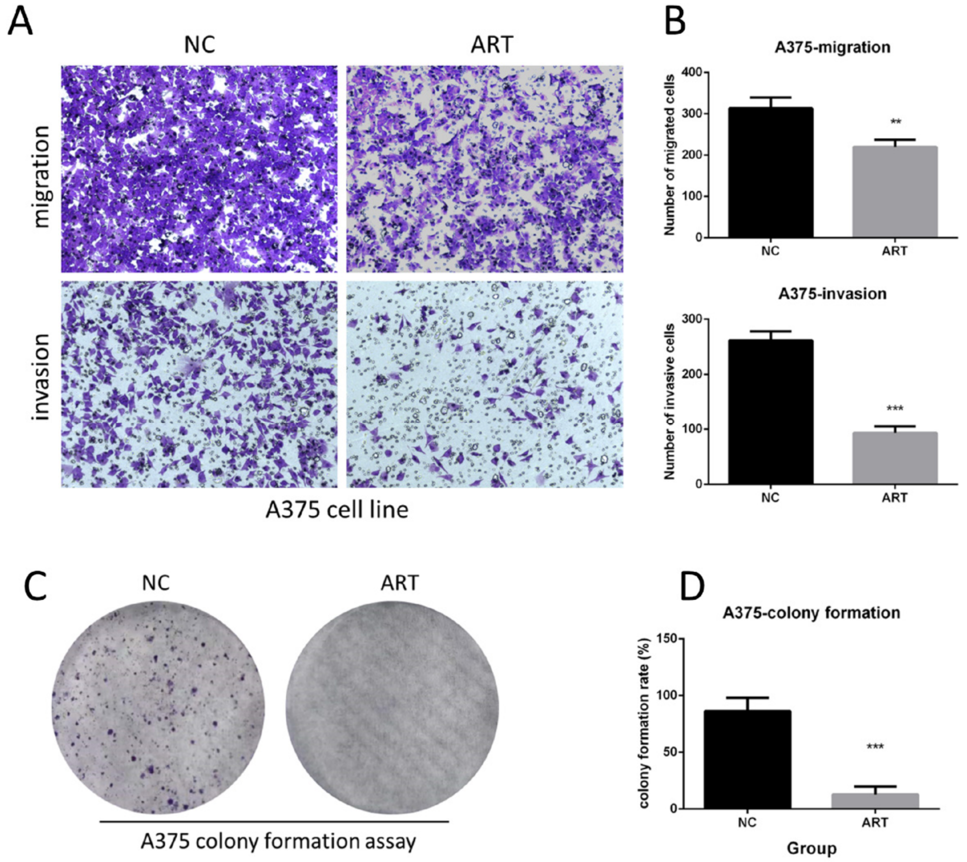

Supplementary figure 2. Artemisinin inhibited proliferation, migration and invasion in A375 cells. A. Transwell migration and invasion assay in A375 cells. B. Statistical result of transwell migration assay and transwell invasion assay in A375 cells. C.Colony formation assay in A375 cells. D. Statistical result of colony formation assay in A375 cell line. *P<0.05, **P<0.01, ***P<0.001.

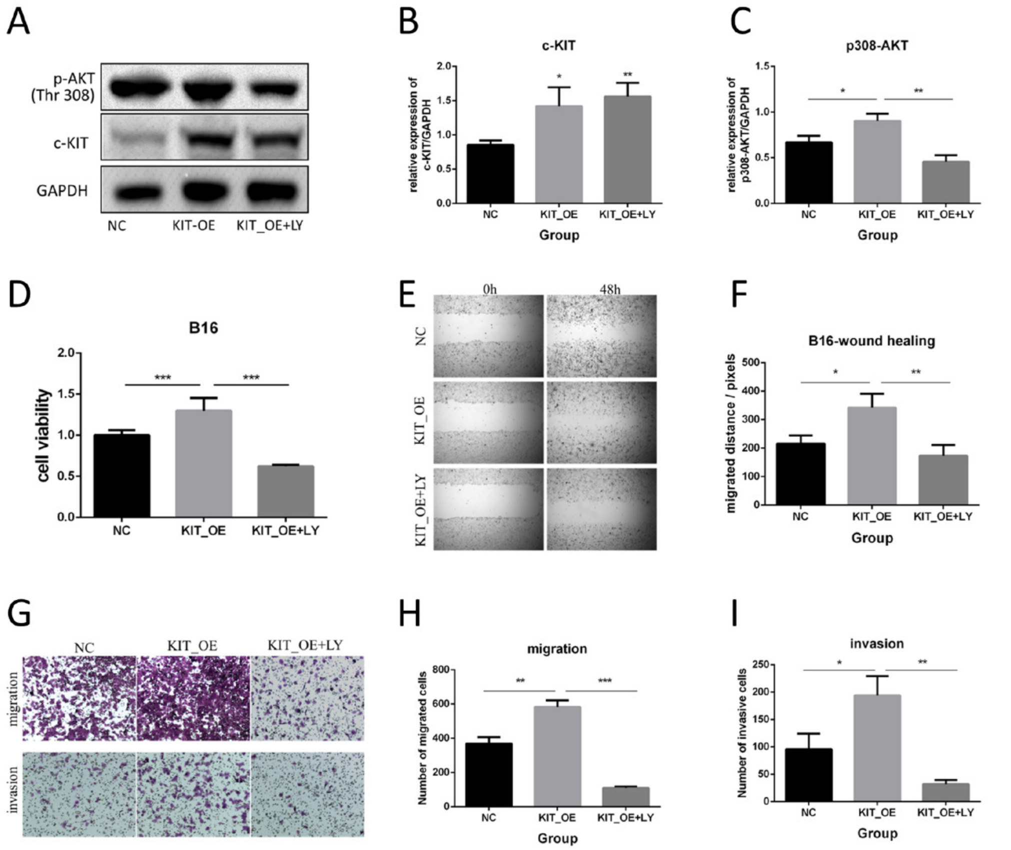

Supplementary figure 3. Overexpression of c-KIT (KIT_OE) promoted melanoma while rescued experiments with LY294002 (LY) reversed the effects. A. Western blot in NC group, KIT_OE group and KIT_OE+LY group. B. Statistical result of the c-KIT relative expression. C. Statistical result of the relative expression of phosphorylated AKT (Thr308). D. MTT assay of the three groups in B16 cells. E. Wound healing assay of the three groups in B16 cells. F. Statistical result of wound healing assay. G. Transwell migration and invasion assay for the three groups in B16 cells. H. Statistical result of transwell migration assay. I. Statistical result of transwell invasion assay. *P<0.05, **P<0.01, ***P<0.001

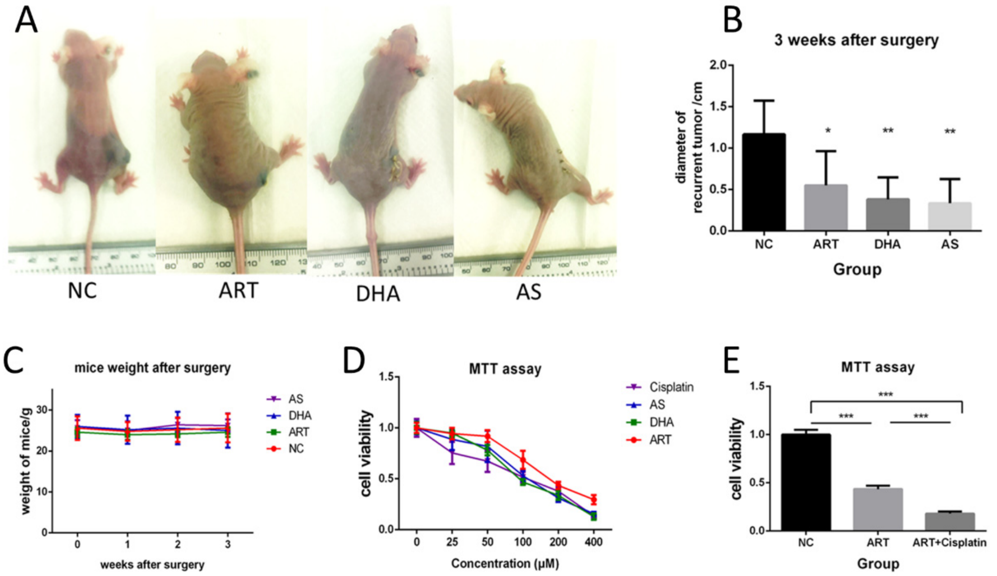

Supplementary figure 4. Comparison between artemisinin (ART) and its deratives dihydroartemisinin (DHA) and artesunate (AS). A. Recurrent melanoma in mice of different groups in 3 weeks after surgery. B. Statistical result of the recurrent melanoma in 3 weeks after surgery. C. The change of mice weight in different groups after surgery. D. MTT assay showed the decreasing trend of cell viability by concentration in ART, DHA, AS and Cisplatin group. E. MTT assay in negative control (NC) group, ART group and ART plus Cisplatin group. *P<0.05, **P<0.01, ***P<0.001
